# "Golden Hour" in Road Traffic Accident Victims: Hurdles and Impact on Mortality

**DOI:** 10.7759/cureus.78772

**Published:** 2025-02-09

**Authors:** Shilpa A Gaikwad, Vaibhav D Shinde, Sachin P Kothavale

**Affiliations:** 1 Emergency Medicine, Bharati Vidyapeeth (Deemed to be University) Medical College and Hospital, Sangli, IND

**Keywords:** arrival, glasgow coma scale, obstacles, outcome, road traffic injuries, traumatic brain injury

## Abstract

Introduction

Road traffic accident (RTA)-related death and disability are alarming health issues globally, which are rapidly increasing in developing countries. Timely presentation to a health center and prompt medical care are needed to prevent mortality and morbidity related to RTAs. This study was conducted to find out the hurdles in the golden hour arrival of RTA victims and to study its impact on RTA mortality at a tertiary health institute.

Materials and methods

This retrospective, cross-sectional analytical study was conducted in the Department of Emergency Medicine of Bharati Vidyapeeth (Deemed to be University) Medical College and Hospital in Sangli, India, over a period of six months from January 1 to June 31, 2022, after obtaining institutional ethical committee approval. This tertiary care center is situated at the border of two states in Western India. All adult RTA victims who were treated during the study period were included. Data were collected using structured, pretested, and validated pro forma. Data was statistically analyzed. The factors associated with mortality were analyzed using a chi-squared test.

Results

We studied a total of 315 RTA victims. Around 49.2% (n=155) of patients were from the 29-48-year age group, and male (87%; n=276) predominance was observed. We observed 46% (n=144) of accidents on weekend days and found alcohol consumption in 45% (n=142). Around 61% (n=193) of accidents occurred within 50 km of our hospital. Only 20.6% (n=65) of patients arrived at a healthcare facility within the golden hour. We did not observe a significant association (p=0.166391) between golden hour arrival and mortality in our study but observed statistically significant associations for mortality with on-arrival Glasgow Coma Scale (GCS) values of less than 8, patients admitted to the intensive care unit (ICU), and the presence of traumatic brain injury. Transport vehicles accounted for 33% (n=104) of the obstacles in reaching the emergency department (ED), followed by finances (30%; n=96), lack of knowledge about the nearby health facility (14.6%; n=46), absence of an accompanying person (11%; n=35), and fear of legal formalities and hospital detention (5%; n=17). Twenty percent (n=63) of patients reported experiencing one or more of these obstacles.

Conclusion

Less than one-fourth of RTA patients arrived at a medical facility during the golden hour. In developing countries like India, the causes of delay are numerous. The severity of injuries determines RTA fatality.

## Introduction

Road traffic accident (RTA)-related morbidity and mortality are major health issues across the world. Road traffic injuries (RTIs) are considered the eighth leading cause of death globally and will become the fifth leading cause by 2030 [1]. Globally, RTI-related deaths account for nearly 1.2 million deaths annually, with an additional 20-50 million injuries or disabilities. These injuries account for 2.1% of global mortality and 2.6% of all disability-adjusted life years (DALYs) lost. RTIs are the leading cause of death in children and young adults aged 5-29 years. Low- and middle-income countries observe 92% of the world's RTI, which significantly impacts local, national, and global economies. Due to rapid urbanization and motorization, RTAs are increasing in developing countries, like India [1-4]. The worst-affected age group among RTAs is the young to middle-aged, which is the socioeconomically active age group [2-4]. The fatality rate from RTAs in India is increasing rapidly [1-4].

Providing pre-hospital treatment and transportation to the most appropriate hospital is crucial to minimize the morbidity and mortality related to RTAs. Late arrival of trauma victims to the hospital is a significant contributing factor to their poor outcomes [5]. The period immediately following the trauma is considered crucial for subsequent outcomes. If RTA victims receive proper medical care within the "golden hour," that is, within the first 60 minutes after the accident, detrimental effects can be minimized [6]. Lack of infrastructure, unawareness about nearby health facilities, poor road access, and financial and communication issues are major hurdles in India for the timely arrival of RTA victims to hospitals [5-7].

Our study aimed to identify the barriers that prevent RTA patients from arriving at the emergency department (ED) during the golden hour and to investigate the correlation between golden hour arrival and in-hospital mortality rates among RTA patients at a tertiary health institution. The study was conducted at a unique geographic location (at the border of two states and also the border of two districts with differences in spoken language, culture, literacy, and economic status) in rural India.

## Materials and methods

Study design and setting

This retrospective, cross-sectional analytical study was carried out in the Department of Emergency Medicine of Bharati Vidyapeeth (Deemed to be University) Medical College and Hospital in Sangli, India, during a six-month period, from January 1 to June 31, 2022, after obtaining institutional ethical committee approval from the institute's Institutional Ethics Committee (approval number: BV(DU)MC&H/Sangli/IEC/521/23).

Methodology

Any accident on a public road with at least one vehicle involved and at least one person injured qualifies as an RTA. After obtaining permission from medical superintendents and approval from the institutional ethical committee, we reviewed all the files of RTA patients who presented at our ED between January 1 and June 31, 2022. All RTA patients above 18 years old, who directly came to our health center (did not receive pre-hospital care or were not treated by other health centers prior to arrival), were studied. The study excluded patients under the age of 18, those who were brought dead, those who were referred from or had previously received treatment at other health centers, those with minor injuries that did not require admission, those who were unwilling to participate in the study, and those who were discharged against medical advice. Patients with incomplete documentation were excluded from the study as well. We collected and entered all the data into a predesigned, validated pro forma.

We divided the data as follows: we recorded the epidemiological profile of patients, including their age, gender, and comorbidity status. We recorded accident details, including the day of the accident and the distance from the accident site and the hospital. We recorded patients' on-arrival Glasgow Coma Scale (GCS) values, the association of alcohol consumption, the presence of traumatic brain injury and treatment areas like wards and intensive care units (ICU), as well as patients' outcomes like discharged or death in hospital. Investigators telephonically interviewed victims or their relatives to gather information about the challenges they faced to reach the hospital following the accident. IBM SPSS Statistics for Windows, Version 22.0 (Released 2013; IBM Corp., Armonk, New York, United States), rechecked the data for completeness and processed it for analysis. We calculated the frequencies and percentages for categorical variables. We analyzed the factors associated with hospital exit outcomes using a chi-squared test. P-values <0.05 were considered statistically significant.

## Results

A total of 746 RTA patients attended the ED during the study period. As per the inclusion criteria, we studied 315 RTA patients.

Most of the RTA victims in our study, i.e., 49.2% (n=155), were in the 29-48-year age group, followed by 30% (n=95) patients in the 49-68-year age group. Our study revealed a male predominance, with 87.5% (n=276) of the accident victims being male and 12.4% (n=39) being female. We observed 46% (n=144) of RTAs on weekend days, i.e., Saturday and Sunday, and 54% (n=177) on weekdays. We found alcohol consumption in 45.1% (n=142) accidents. Around 61.3% of accidents had happened within 50 km of our hospital, while 39% of accidents had happened beyond 50 km. P<0.05 was considered statistically significant. Variables such as age, gender, alcohol consumption, day of the accident, and distance from the accident site and hospital did not show statistical significance with in-hospital mortality (Table 1).

**Table 1:** Sociodemographic data (gender, age), alcohol consumption (yes/no), day of the accident (WE/W), and hospital accessibility (<50 km/>50 km) WE: weekend; W: weekdays; n: number (frequency)

Variable		Total	Discharged	Death	P-value
Gender	Male	276 (87.6%)	161 (58.3%)	115 (42.1%)	0.20296
	Female	39 (12.4%)	25 (64.1%)	14 (36.4%)	
Age (years)	18-28	53 (16.8%)	26 (49.1%)	27 (51.4%)	0.58093
	29-48	155 (49.2%)	93 (60%)	62 (40.3%)	
	49-68	95 (30.2%)	61 (64.2%)	34 (35.4%)	
	69 and above	12 (3.8%)	6 (50%)	6 (49.7%)	
Alcohol consumption	Yes	142 (45.1%)	75 (47.2%)	67 (52.8%)	0.092
	No	173 (54.9%)	173 (54.9%)	62 (64.2%)	
Day of the accident	WE	144 (45.7%)	83 (57.6%)	61 (41.9%)	0.140042
	W	171 (54.3%)	103 (60.2%)	68 (39.3%)	
Hospital	Remote	122(38.7%)	69 (56.6%)	53 (43.8%)	0.73797
	Accessible	193(61.3%)	117 (60.6%)	76 (39.8%)	

We observed that 42% (n=131) of victims had a GCS value of less than 8, whereas 14% (n=43) had a GCS value ranging from eight to 12. The remaining 46% (n=141) of patients had a GCS value of more than 12. We observed that 58% (n=184) of patients had a traumatic brain injury, while 31% (n=131) did not. Due to the severity of their injuries as per GCS and the presence of traumatic brain injuries, 65% (n=205) of patients were in the ICU, while 35% (n=110) received treatment in the ward. We noted comorbidities such as hypertension, diabetes, and chronic obstructive pulmonary disease (COPD) in 47% (n=147) of patients, while 53% (n=168) did not have any comorbidity. Statistically, correlations were observed between on-arrival GCS values of less than 8, the admission of patients to the ICU, and the presence of traumatic brain injury (Table 2).

**Table 2:** GCS, treating area (ICU/W), injury (isolated traumatic injury/traumatic brain injury with other injuries such as blunt/penetrating or extremity injuries/no evidence of traumatic brain injury), and number of patient's comorbidities GCS: Glasgow Coma Scale; ICU: intensive care unit; W: ward; n: number (frequency)

Variable		Total	Discharged	Death	P-value
GCS	<8	131 (41.6%)	33 (25.2%)	98 (75.3%)	<0.00001 (significant association)
	8-12	43 (13.7%)	21 (48.8%)	22 (51%)	
	>12	43 (13.7%)	132 (93.6%)	9 (6.5%)	
Treating area	ICU	205 (65.1%)	80 (39%)	125 (61.2%)	<0.00001 (significant association)
	W	110 (34.9%)	106 (96.4%)	4 (3.6%)	
Injury	Traumatic brain injury	106 (33.7%)	46 (43.4%)	60 (56.3%)	<0.00001 (significant association)
	Traumatic brain injury and other injuries	78 (24.8%)	24 (30.8%)	54 (69.4%)	
	No traumatic brain injury	131 (41.6%)	116 (88.5%)	15 (11.3%)	
Comorbidity	0	168 (53.3%)	92 (54.8%)	76 (45.3%)	0.24786 (no significant association)
	1	70 (22.2%)	45 (64.3%)	25 (35.3%)	
	2 and more	77 (24.4%)	49 (63.6%)	28 (36.7%)	

The percentage of RTA victims who reached the tertiary health center within the golden hour is shown in Table 3. Only 20.6% (n=65) of the 315 patients reached the health center within the golden hour, while 79.4% (n=250) arrived after 60 minutes of the accident. About 53.8% (n=35) of patients in the golden hour arrival group were discharged to home, and in-hospital mortality was observed in 46.2% (n=29). Among the patients who arrived at the hospital after the golden hour, 60.4% (n=151) of patients were discharged, while the mortality rate was 39.8% (n=99). We did not observe a significant association (p=0.166391) between golden hour arrival and in-hospital mortality in RTA victims in our study (Table 3).

**Table 3:** Arrival time after the accident n: number (frequency)

Variable		Total	Discharged	Death	P-value
Arrival time	<1 hour	65 (20.6%)	35 (53.8%)	30 (46.2%)	0.166391 (no significant association)
	>1 hour	250 (79.4%)	151 (60.4%)	99 (39.8%)	

Our study did not observe a statistically significant association between in-hospital mortality and variables such as age, gender, day of the accident, alcohol consumption, hospital accessibility, and existing comorbidities. Variables such as on-arrival GCS values, the association of traumatic brain injury, and patient treatment in the ICU showed statistically significant mortality. We considered on-arrival GCS values as a severity marker. We observed 75% in-hospital mortality for RTA victims who arrived with GCS values <8 and that 93% of RTA victims with on-arrival GCS values >12 were discharged to home.

Analysis of hurdles or difficulties for reaching the ED within the golden hour revealed that 104 (33%) patients encountered transport vehicle-related issues, 96 (30.5%) patients had financial problems, 35 (11.11%) patients did not have an accompanying responsible person, 42 (13.3%) patients faced communication issues due to different languages spoken in adjacent states, 46 (14.6%) patients did not know the nearest or affordable health facility, and 17 (5.3%) patients were brought late by relatives or friends due to fear about legal formalities and detention at hospitals. We identified 63 (20%) patients who experienced multiple delays in arriving (Table 4).

**Table 4:** Hurdles RTA victims faced to reach the hospital RTA: road traffic accident; n: number (frequency)

Variable	Category	Frequency (percentage)
Hurdles	Vehicle-related	104 (33%)
	Financial issues	96 (30.5%)
	Non-availability of a responsible person	35 (11.11%)
	Fear about legal formalities and detention at hospitals	17 (5.3%)
	Language	42 (13.3%)
	Unawareness about nearby health facilities	46 (14.6%)
	Mixed	63 (20%)

## Discussion

Researchers have conducted numerous studies on various aspects of RTAs. This study is distinguished by the unique geographic location of its study center. Our health facility is uniquely located in rural India, straddling the borders of two states, Maharashtra and Karnataka, and also two districts of the Maharashtra state. There are multiple differences regarding language (Maharashtra language is Marathi and Karnataka language is Kannada), economy, and literacy levels in the residing population between the two adjacent states. 

This study examined a total of 315 patients. The present study observed a predominance of young age group and male gender involvement in RTA. We observed 42% mortality in the 18-48-year age group. These findings are in accordance with other studies reported across the world [1-8]. This study observed 54.9% of RTAs on weekdays, that is, from Monday to Friday. Alcohol consumption was noted in 45.1% of accident victims, and 52.8% mortality was observed in victims who consumed alcohol. Alcohol was evidenced as one of the potential risk factors for fatal injuries due to accidents in multiple studies [3,9,10].

R Adams Cowley introduced the concept of "golden hour," referring to the initial 60 minutes following a traumatic injury, which is crucial for reducing mortality and morbidity in trauma victims. Delay in arriving at the nearest trauma center is the major obstacle to the "golden hour" treatment for RTA victims. In the current study, 20.6% of RTA victims arrived at the ED within the first 60 minutes of the accident. In our study, the hospital was accessible from the accident site in 61.3% of accidents. Antony et al. [6] found that 55% of RTA victims arrived at healthcare facilities within an hour, with an average arrival time of three hours (IQR 1.3-5.1 hours). A study conducted in Israel by Tiruneh et al. [11] reported a higher percentage due to the faster and more professional evacuation of persons injured in RTA. The median arrival time of RTA victims at the health facilities was 8±20 hours, accounting for 72.61% of those who were referred from other health facilities as reported in a retrospective study by Afacho et al. [12]. The arrival time for RTA patients is different in developed and developing countries. Prompt dispatch to the nearest health center, established trauma center, and effective pre-hospital care are major components of developed and developing countries. The arrival time for RTA patients varies in both rural and urban areas due to various factors such as population fluctuations, undeveloped road infrastructure, varying levels of education and economy, and the unavailability of health facilities.

Our study identified several obstacles to accessing the health center, including transportation issues (33%), financial concerns (30.5%), lack of knowledge about nearby health facilities (14.6%), language barriers (13.3%), lack of a responsible companion (11.11%), and fear of legal formalities (5.3%). We observed that 20% of patients had more than one difficulty for arrival to a health facility. It would be helpful to display bilingual boards for awareness about the nearest health center and free government ambulance services at unique geographical locations like our study place. 

Antony et al. [6] identified communication delays, vehicle arrival delays, and prolonged transport periods as the primary causes of delays in reaching healthcare facilities. The most common major hurdles in India for the timely arrival and prompt post-trauma care of RTA victims are infrastructure, road network, unawareness, and economic and communication issues [5-7]. Unavailability or underutilization of pre-hospital emergency services in developing countries, like India, was attributed to increased mortality, whereas in developed countries, trauma-related morbidity and mortality are reduced due to prompt and appropriate pre-hospital care [5-7].

We did not observe a statistically significant association (p=0.166391) between arrival time and in-hospital mortality in the present study. Afacho et al. [12] also reported a high death rate in the early arrival group. However, Antony et al. [6] found better outcomes for RTA victims who arrived within the golden hour as well as a statistically lower mortality rate for those who directly arrived at the tertiary care facilities. Chandrasekharan et al. [13] reported similar findings, stating that delays in transfers of patients and a lack of pre-hospital emergency services were significantly associated with increased mortality. A study conducted by Mishra et al. [14] in 2010 in West Nepal, India, revealed that 10% of patients were admitted to the hospital within an hour. Bigdeli et al. [15] stated that the emergency medical services (EMS) response and transport of RTA victims indirectly affect the "golden hour" for victim management.

In our study, the mortality rate was significantly higher among RTA victims who had an on-arrival GCS value of less than 8, had a traumatic brain injury, and required ICU care. Afacho et al. [12] also provided evidence supporting these findings. They observed a fourfold higher mortality in ICU-admitted patients and attributed a sixfold increase in mortality to GCS values of less than 8. RTA patients with mild and moderate GCS scores predict a better outcome than those with severe GCS scores. Injury severity score, trauma score, and GCS score are considered to assess the severity of traumatic injuries. The presence of severe head injury, polytrauma, and hemodynamic instability are risk factors for trauma-related mortality [7,12,15,16]. Mitra et al. [17] also found no relationship between out-of-hospital time and outcome in trauma victims. Newgard et al. [18] evaluated and reported no association between EMS intervals and mortality among trauma patients with field-based physiologic abnormality. These findings are in accordance with our findings. Hence, patients arriving within the golden hour but with severe injuries leading to hemodynamic instabilities would be the probable explanation for high mortality in this group. 

This is the first study conducted at a unique geographic location (at the border of two states and also the border of two districts with differences in spoken language, culture, literacy, and economic status) in rural India to find hurdles in the golden hour arrival of RTA victims and also to study the impact of golden hour arrival on RTA. However, the study was limited with regard to assessing the severity of injuries by using injury severity score and also road condition, pre-hospital care, and hospital care standards received by RTA victims. 

## Conclusions

We conducted this study at a tertiary health institute in rural India, spanning two states. Around 20.6% of patients arrived within the first 60 minutes after an RTA in our study, despite 61.3% of these accidents having happened within 50 km of our hospital. Our study identified transport vehicles, finances, communication, awareness about nearby health facilities, fear of legal formalities, and the availability of attendants as barriers to reaching health facilities after RTA.

We found no significant correlation between golden hour arrival and in-hospital mortality in RTA patients. The severity of traumatic injuries, on-arrival GCS values of less than 8, the presence of brain injury, and critical conditions requiring intensive medical care were observed as impacting factors for RTA victims' in-hospital mortality.
